# Adenosine A_2A_ Receptors in Psychopharmacology: Modulators of Behavior, Mood and Cognition

**DOI:** 10.2174/157015909789152191

**Published:** 2009-09

**Authors:** Hai-Ying Shen, Jiang-Fan Chen

**Affiliations:** 1 Robert Stone Dow Neurobiology Laboratories, Legacy Research, Portland, OR 97232, USA; 2 Department of Neurology, Boston University School of Medicine, Boston, MA 02118, USA

**Keywords:** Adenosine, A_2A_ receptor, caffeine, psychostimulant, amphetamine, cocaine, schizophrenia, anxiety, depression, dopamine, glutamate.

## Abstract

The adenosine A_2A_ receptor (A_2A_R) is in the center of a neuromodulatory network affecting a wide range of neuropsychiatric functions by interacting with and integrating several neurotransmitter systems, especially dopaminergic and glutamatergic neurotransmission. These interactions and integrations occur at multiple levels, including (1) direct receptor- receptor cross-talk at the cell membrane, (2) intracellular second messenger systems, (3) trans-synaptic actions *via *striatal collaterals or interneurons in the striatum, (4) and interactions at the network level of the basal ganglia. Consequently, A_2A_Rs constitute a novel target to modulate various psychiatric conditions. In the present review we will first summarize the molecular interaction of adenosine receptors with other neurotransmitter systems and then discuss the potential applications of A_2A_R agonists and antagonists in physiological and pathophysiological conditions, such as psychostimulant action, drug addiction, anxiety, depression, schizophrenia and learning and memory.

## INTRODUCTION

Behavior, mood and cognition were previously considered to be mainly controlled by dopaminergic and glutamatergic neurotransmission. The ability of the adenosinergic system to modify these behaviors and cognitive function has attracted a great deal of attention as increasing evidences support the tight relationship between adenosine-based modulation and the dopaminergic and glutamatergic systems. Adenosine is ubiquitously distributed throughout the central nervous system (CNS). While early research pointed to the role of adenosine as a metabolite of adenosine triphosphate (ATP) and cyclic adenosine monophosphate (cAMP), the importance of this molecule is now widely recognized as a modulator of neurotransmission and complex behaviors. Indeed, adenosine fulfills two important roles: (1) as a homeostatic transcellular messenger in all cells; (2) and particularly as a neuromodulator controlling neurotransmitter release and neuronal excitability [31, 63].

Endogenous extracellular adenosine, acting mainly through adenosine A_1_ and A_2A_ receptors (A_1_Rs and A_2A_Rs) in the CNS, controls and integrates a wide range of brain functions, most notably regulation of sleep, locomotion, anxiety, cognition and memory [47, 63, 64]. Consequently, dysfunction of adenosinergic signaling is implicated in pathologies ranging from epilepsy to neurodegenerative disorders to psychiatric conditions [175]. Owning to adenosine’s unique role of integrating glutamatergic and dopaminergic neurotransmission systems, adenosine-based therapies are rapidly evolving in preclinical and clinical studies for the treatment of different neurological disorders [62] and the adenosinergic system is increasingly recognized as a potential target for the development of new therapies for psychiatric disorders [32, 34].

The distribution, molecular structure and function of A_2A_Rs in the brain, has extensively been reviewed elsewhere [22, 32, 63, 65]. Briefly, the A_2A_R belongs to the G-protein coupled adenosine receptor family and is highly expressed in the striatum [61, 182]. A_2A_Rs are also expressed at lower levels in other brain areas, including hippocampus, cerebral cortex, nucleus tractus solitarius, motor nerve terminals and glial cells. Activation of A_2A_Rs enhances the release of several neurotransmitters, such as acetylcholine, glutamate and dopamine, but inhibits gamma aminobutyric acid (GABA) release [24, 36, 47, 109]. A_2A_R activation also modulates neuronal excitability and synaptic plasticity, and affects various behaviors including locomotor activity, sleep-wake cycle, anxiety, depression and learning and memory. At the cellular level, A_2A_Rs are localized predominantly in the soma of GABAergic (enkephalin-containing, dopamine D_2_ receptor-expressing) striato-pallidal projection neurons and to a lesser extent in asymmetrical excitatory synapses at the dendrites of cortico-striatal terminals [7, 61, 163, 182, 203]. At the molecular level, the A_2A_R has been shown to interact with other neurotransmitters and neuromodulator receptors (possibly through molecular dimerization), including dopamine D_2_ receptor (D_2_R), adenosine A_1_ receptor (A_1_R), cannabinoid CB1 receptor (CB1R), metabotropic glutamate receptor subtype 5 (mGluR5) and facilitatory nicotinic acetylcholine (Ach) receptor. These interactions expand the range of possibilities used by adenosine to interfere with neuronal function and communication [47, 57, 59, 181].

In the present overview, we mainly recapitulate some molecular features of brain A_2A_Rs and the ability of the A_2A_R to integrate several neurotransmission and signaling pathways that might be relevant to the potential therapeutic interest of psychopharmacology, particularly in psychostimulation, drug addiction, anxiety, depression, psychiatric disorder, e.g. schizophrenia, and in learning and memory [185].

## MOLECULAR BASIS FOR A_2A_R MODULATION OF OTHER NEUROTRANSMITTER SYSTEMS IN THE BRAIN 

	A_2A_Rs are highly expressed in the striatum, a pivotal locus with high levels of neurotransmission and neurotransmitter receptors, thus providing an anatomical basis for the interaction between the A_2A_R and other, such as dopaminergic and glutamatergic, neurotransmitter systems. These interactions occur at multiple levels, including (1) direct receptor-receptor cross-talk at the cell membrane, (2) intracellular second messenger systems, (3) trans-synaptic actions *via* striatal collaterals or interneurons in the striatum, (4) and interactions at the network level of the basal ganglia. Compared to other relatively “circumlocutory” interactions, the intramembrane receptor-receptor interactions are more direct in spacial connection. Agnati and Fuxe first reported experimental observations for the existence of membrane receptor-receptor interaction [1, 70, 221]. Since then, the concept has been further developed to receptor-receptor heteromers and the so called “receptor mosaic” with the discovery of aggregates of multiple receptors [67, 71]. The interactions involving the A_2A_Rs have been described for several G-protein coupled receptors, including D_2_Rs, A_1_Rs, CB1R and mGluR5 [54, 181]. The largely antagonistic and occasionally synergistic interactions between the A_2A_R and other receptors occurring directly between receptor complexes have been documented.

### 1. Interaction Between Adenosine A_2A_Rs and A_1_Rs

	The prevalent neuromodulatory influence of adenosine is inhibitory on neuronal activity in the brain [63]. Adenosine is known to modulate the release of many neurotransmitters, including dopamine, glutamate, GABA, serotonin, noradrenaline and ACh, though the inhibition of excitatory neurotransmitters (e.g. glutamate) is most pronounced [31, 47, 66]. Adenosine modulation of neurotransmitter release is mediated through the activation of the A_1_R and A_2A_R. Adenosine activation of G_i_-coupled A_1_Rs reduces neurotransmitter release at pre-synaptic nerve terminals and depresses neuronal firing at postsynaptic sites [66, 121, 194]. In contrast, adenosine activation of the G_s/olf_-coupled A_2A_R has been demonstrated to exert an excitatory modulation on the neurotransmitter release of glutamate and ACh in the striatum, and ACh in the hippocampus [33, 110]. The A_2A_R also controls GABA release in the striatum [108] as well as in the hippocampus [185]. Additionally, the activation A_2A_R decreases the functionality of the A_1_R in some experimental settings [46, 130, 131, 155].

	A_1_Rs and A_2A_Rs may be activated under different conditions; adenosine may preferentially act at A_1_Rs under basal condition, probably due to its relatively high expression level and wide-spread distribution in the brain [31, 63]. However, the different affinities of adenosine for A_1_Rs and A_2A_Rs is still an open issue [35, 47]. It has been suggested that A_1_Rs largely maintain tonic homoeostatic adenosine functions whereas A_2A_Rs mostly exert its fine-tuning modulation under some pathophysiological situations [170, 186]. Such receptor discrimination may be achieved through the pattern of neuronal firing (i.e. with high neuronal discharge, there may be higher levels of ATP and adenosine in the synapse), the different sources of adenosine (i.e. intra- and extracellular formation), the localization of relevant synthetic or metabolic enzymes, or the relative position of adenosine release and receptor sites (synaptically versus extra-synaptically) [35, 94, 174, 184]. Furthermore, the partially overlapping distributions of these two adenosine receptors may also permit local formation of heteromers to exert their opposite modulating effects directly *via* a so called "concentration-dependent switch" mechanism [54].

### 2. Interaction Between Adenosine A_2A_Rs and Dopamine D_2_Rs

	Striatal A_1_Rs and A_2A_Rs are major neuromodulator receptors that exert profound effects on D_1_Rs- and D_2_R-mediated dopamine signalling and function in the striatum. Evidence suggests the existence of antagonistic A_1_R-D_1_R heteromeric receptor complexes in the basal ganglia and prefrontal cortex, particularly in the direct striatonigral GABA pathways. The antagonistic A_1_R-D_1_R interactions at the neurochemical and behavioral levels can be explained in part by the existence of such A_1_R-D_1_R heteromeric receptor complexes and by antagonistic interactions at the level of the second messengers. On the other hand, A_2A_R-D_2_R heteromers have been demonstrated as the first example of epitope-epitope electrostatic interactions underlying receptor heteromerization [55]. A large number of studies with different techniques, i.e. coimmunoprecipitation, fluorescence resonance energy transfer (FRET), bioluminescence resonance energy transfer (BRET), biochemical binding and signaling, microdialysis and behavioral pharmacology have indicated the existence of A_2A_R-D_2_R heterodimers in the striato-pallidal GABA neurons, where activation of A_2A_Rs reduces binding, coupling and signaling of D_2_Rs [18, 23, 52, 91, 207]. However, since supporting evidence from *in vivo* co-immunoprecipitation studies could be subjected to other interpretations, the evidence of A_2A_R-D_2_R dimmers in intact brain tissues is still not clear yet. Further studies are needed to conclusively demonstrate the functional significance of receptor heterodimer *in vivo*.

	The antagonistic A_2A_R-D_2_R interactions in brain have also been demonstrated at the second messenger levels [146, 183, 199], through which the A_2A_R strongly modulates the excitability in the striato-pallidal GABA neurons probably *via* its ability to counteract D_2_R signaling to multiple effectors. For example, the activation of the A_2A_R can counter the D_2_R-induced inhibition of the Ca^2+^ influx over the L-type voltage-dependent Ca^2+^ channels (CAV 3.1 channels) *via* the activation of phospholipase C and protein phosphatase-2B [54, 90]. The counteraction of this cascade by A_2A_Rs may involve G_o_ and/or G_q11_ protein with release of the βγ subunits, and leads to increased phosphorylation of CAV3.1 channels and favoring an upstate of the striatal neuronal firing [200]. Furthermore, the D_2_R-induced reduction of firing rates in the dopamine-denervated striatum is enhanced by A_2A_R antagonists and attenuated by A_2A_R agonists [199]. There also exists a reciprocal interaction between A_2A_R-D_2_R receptors, through which the activation of D_2_Rs can inhibit the A_2A_R-induced increase in cAMP accumulation *via* G_i/o_ at the level of adenylate cyclases [54, 69, 114]. 

### 3. Interaction Between Adenosine A_2A_Rs and Dopamine D_1_Rs

	Pharmacological studies have revealed functional interaction between A_2A_R and D_1_R [144, 145, 162, 163]. At the systemic level, pharmacological blockade of A_2A_R potentiates D_1_R agonist-induced rotational behavior [145] and c-fos expression in dopamine-depleted striatum [162]. The modulation of phosphorylation on neuronal dopamine and cAMP-regulated phosphoprotein 32 (DARRP-32) by the interaction of A_2A_R and D_1_R has been investigated in brain slices and intact animals [85, 205]. DARPP-32 is expressed in the medium spiny neurons of both the direct and indirect pathways. Stimulation of D_1_Rs and A_2A_Rs or blockade of D_2_Rs increases DARPP-32 phosphorylation in distinct cell populations of the striatum [204]. Blockade of A_2A_Rs or stimulation of D_2_Rs not only abolishes D_2_R antagonist- or A_2A_R agonist-induced DARPP-32 phosphorylation, but also antagonizes the D_1_R agonist-induced DARPP-32 phosphorylation in the striatum [201]. Furthermore, tetrodotoxin (TTX) blocks this A_2A_R-D_1_R interaction, suggesting a trans-synaptic (network) cross-talk between A_2A_Rs and D_1_Rs [127]. Importantly, DARRP-32 can integrate two distinct pathways, adenosine and dopamine signaling, thus providing a possible molecular explanation for the long-known behavioral interaction between A_2A_Rs and D_1_Rs [63]. Lindskog *et al.* (2002) suggested that DARPP-32 is required for A_2A_R inhibition-induced persistent motor stimulation since caffeine-induced motor activity is greatly reduced in DARPP-32 knockout mice [128]. At the molecular level, caffeine treatment reduces phosphorylation of DARPP-32 at the Thr34 site by blocking A_2A_Rs [201]. Conversely, caffeine increases phosphorylation of DARPP-32 at the Thr75 site *via* an inhibitory feedback loop of protein kinase A (PKA), which leads to further reduction of PKA activity through feedback inhibition [14, 128, 152]. Thus, DARPP-32 appears to be an important molecular target for integration of adenosine and dopamine signaling through phosphorylation at Thr34 and Thr75 sites.

	Recently, a genetic study of drug addiction showed that D_1_R-A_2A_R double knockout mice shared phenotypic similarities of some behavioral components with A_2A_R knockout mice or the mice with sole deficiency of D_1_Rs in terms of preference for ethanol and saccharin; whereas other components of behavioral phenotypes in the D_1_R-A_2A_R double knockouts were likely attributable to the loss of both receptors [191]. These data suggest an interaction of D_1_Rs and A_2A_Rs in the reinforcement processes underlying the intake of rewarding substances. In addition, there is limited evidence for the interaction of A_2A_R and D_3_R [210].

### 4. Interaction Between A_2A_Rs and Glutamatergic Neurotransmission

	Glutamate is the main excitatory neurotransmission in the CNS. Glutamate activates either ionotropic receptors (including NMDAR, AMPAR and kainate-type receptor) that are mostly localized in the postsynaptic density [12] or G protein-coupled metabotropic glutamate receptors (mGluRs) that are mostly localized extrasynaptically [209]. A_2A_Rs interact with glutamatergic system at several levels in the brain. First, ultrastructural findings suggest that extra-striatal A_2A_Rs are mostly synaptically-located [171], particularly in glutamatergic synapses [173]. These presynaptic A_2A_Rs have been demonstrated to control the release of glutamate in the striatum, cerebral cortex and hippocampus [24, 130, 134, 164] and NMDAR activity in the striatum [172]. Second, it has been reported that A_2A_Rs may indirectly control the level of extracellular glutamate by modulating the activity of glutamate transporter in astrocytes [72, 154].

	Third, the receptor heterodimer mechanism is also suggested to underline the interaction of A_2A_R and the glutamatergic system, particularly with mGluR. The immunoreactivities of A_2A_R and mGluR5 were found to be colocalized in primary cultures of striatal neurons [68] as well as in striatal glutamate nerve terminals [177]. Furthermore, coimmunoprecipitation studies suggest that the existence of possible heteromeric receptor complexes containing A_2A_R and mGluR5, where synergism may occur between A_2A_R and mGluR5 [57]. This heteromeric receptor complex is believed to underlie the finding that agonists of A_2A_R and group I mGluR could synergistically reduce the affinity of D_2_R agonist binding sites in striatal membranes [58].

	Fourth, concurrent stimulation of A_2A_R and mGluR5 results in synergistic interactions at the level of c-fos expression and phosphorylation of extracellular signal-regulated kinases (ERK) and DARPP-32 in the striatum [57, 153]. Combined A_2A_R and mGluR5 activation have also led to synergistic cellular effects on GABA release in the ventral striato-pallidal GABA neurons [44]. Recently, Coccurello *et al.* (2004) first demonstrated a synergism between A_2A_R and mGluR5 in the control of locomotion [25], which provides a direct functional link between A_2A_R and the glutamatergic system and also strengthens the A_2A_R as potential target to modulate psychostimulant effects. In addition, a study from Schwarzschild’s group (2005) demonstrated that co-administration of the selective mGluR5 antagonist MPEP and selective A_2A_R antagonist KW-6002 exerts synergistic locomotor stimulation in both normal and Parkinsonian mice [98]. The dependence of MPEP-induced motor activity on the A_2A_R and mGlu5R further demonstrates the functional interaction between A_2A_R and mGluR5 at the behavioral level.

## PSYCHIATRIC BEHAVIORAL EFFECTS AND THERAPEUTIC POTENTIAL OF A_2A_R MANIPULATION IN THE BRAIN

### 1. Psychostimulant Effects

	A_2A_Rs in medium spiny neurons have been established to be the determinant for the control of motor function, since A_2A_R ligands produce most significant motor effects [63, 64, 181, 183] that were abolished in mice deficient in A_2A_Rs [126]. In fact, A_2A_R modulation of normal or hyperdopaminergic conditions is relevant to psychopharmacology [117, 118], whereas the A_2A_R control of the hypodopaminergic condition is directly relevant to Parkinson’s disease (PD) therapy [38, 180]. In dopamine depleted animals, the main mechanism by which A_2A_R antagonists improve motor activity is proposed to be *via* modulation of GABA release. Thus, the systemic administration of A_2A_R antagonists increase motor activity in animals pretreated with D_2_R antagonists, reserpine, 6-OH-dopamine or after genetic inactivation of D_2_R [21, 88, 102, 161, 190, 214] or MPTP-treated monkeys [86, 103].

	On the other hand, the antagonistic interaction of A_2A_R-D_2_R is considered to be the basis for the potential therapy of neuropsychiatric disorders. A_2A_R agonists inhibit, and A_2A_R antagonists potentiate the motor, discriminative, and rewarding effects of psychostimulants [60, 89, 97, 111, 160, 165, 176, 189]. The non-selective A_1_R and A_2A_R antagonist, caffeine, also potentiates these effects of psychostimulants [26, 73, 74, 143, 147]. Intriguingly, genetic inactivation of global A_2A_R or A_2A_R in forebrain neurons has been shown to attenuate acute psychostimulant effects as well as psychostimulant behavioral sensitization [10, 20, 21]. To provide an explanation for the well-known discrepancies - pharmacological blockade and genetic deletion of A_2A_R potentiates and attenuates, respectively, psychostimulant effects [10, 20, 21, 60], we recently showed that the selective inactivation of striatal A_2A_Rs enhances the psychostimulant effect while inactivation of forebrain (including striatal, cortical and hippocampal) A_2A_Rs attenuate psychostimulant effects. This study suggests that striatal A_2A_R and extra-striatal A_2A_R offer opposite modulation, possibly through different effects of pre- and post-synaptic A_2A_Rs in the striatum [188].

	Furthermore, a recent coimmunoprecipitation study demonstrates that A_2A_Rs are able to form receptor complexes with CB1R in the rat striatum, where they are colocalized in dendritic processes and possibly nerve terminals [19]. Thus, the function of CB1R is apparently dependent on A_2A_R activation and modulation of A_2A_Rs may affect the rewarding behavior of cannabinoid [6, 219]. In fact, the finding of A_2A_R-mediated glutamate release and A_2A_R-CB1R interaction in the striatum opens up an interesting possibility of A_2A_R-based psychopharmacological therapy.

### 2. Drug Addiction

	Drugs of abuse have varying mechanisms of actions that create complex behavioral patterns related to drug consumption, drug-seeking, withdrawal and relapse. The extracellular levels of adenosine are elevated upon exposure to drugs of abuse [9] and may modify addiction-related behavior [16]. By acting at the A_2A_R in the ventral striatum, modulation of A_2A_R activity may influence the reinforcement processes underlying opiate, ethanol and psychostimulant intake [17].

	For example, the facilitative role for the A_2A_R has been suggested in opiate reward, reinforcement as well as opiate-seeking behavior. The A_2A_R agonist CGS 21680 increases, while the A_2A_R antagonist DMPX reduces, morphine self-administration in rats [178]. Recently, using A_2A_R knockout mice, Soria *et al.* (2006) showed that A_2A_R knockout mice display a lower rate of cocaine self-administration, a reduction in the maximal effort to obtain a cocaine infusion, and a vertical shift of the cocaine dose-response curve [196]. This indicates that A_2A_Rs seem to be required to develop the addictive effects of this drug. Furthermore, decreased morphine self-administration, breakpoint and conditioned place preference were also observed in A_2A_R knockout mice [17], These data support a decrease in motivation of morphine consumption, perhaps reflecting diminished rewarding effects of morphine, in A_2A_R knockout mice. The mechanism underlying attenuated reward behavior of A_2A_R knockout mice is not clear, but these findings are consistent with previous studies showing a synergistic rather than an antagonistic D_2_R-A_2A_R interaction [16]. Furthermore, a dysregulation of glutamatergic signaling caused by inactivation of presynaptic A_2A_R could be partially responsible for this phenotype. This is in line with the notion that molecular adaptations of the cortico-accumbens glutamatergic synapses are involved in compulsive drug seeking and relapse.

	However, A_2A_R inactivation may play a differential role in the modulation of psychostimulant effects, depending on the involvement of either striatal A_2A_Rs located on the medium spiny neurons themselves, or A_2A_Rs located on the cortical glutamatergic afferents that synapse on these striatal neurons [188]. On one hand, the activation of A_2A_Rs can positively modulate glutamatergic input to the nucleus accumbens through synergistic interactions with mGluR5, and thus maintain a facilitative role in behavior such as psychomotor sensitization and addictive behavior as described above. Alternatively, through antagonistic interaction with D_2_Rs, activation of A_2A_Rs can attenuate the rewarding effects of psychostimulant drugs. Indeed, in the study of reinstatement of cocaine-seeking behavior [215], the A_2A_R antagonist CGS15943 was found to reinstate cocaine-seeking and functions as an intravenous reinforcer, while the A_2A_Rs agonist CGS21680 was found to produce a rightward shift in the CGS15943 reinstatement dose-effect curve. Thus, it remains to be determined whether A_2A_R influences reward *via* striatal A_2A_Rs or extra-striatal A_2A_Rs. It is also possible that A_2A_Rs may either directly interact with the reward (i.e. dopamine or opioid) system or indirectly *via* interaction with other neurotransmitter systems such as glutamate or cannabinoids in the brain.

### 3. Anxiety

	Clinical investigations, pharmacological studies and models of genetically modified rodents have implicated adenosine receptors in the etiology and modulation of various types of anxiety. Caffeine and alcohol have been involved in anxiety-related behavior, due to their antagonism at adenosine receptors and ability to increase adenosine levels, respectively. The adenosine effects on anxiety have been partly attributed to the anxiogenic effects of A_1_R antagonism. However, there are several lines of evidence indicating the involvement of the A_2A_R in anxiety. First, spontaneous anxiety-like behavior is enhanced in A_2A_R knockout mice compared to their WT littermates [13, 15, 122], indicating that adaptive mechanisms in A_2A_R knockouts may result in increased propensity for anxiety. Second, human genetic association studies indicate the association between A_2A_R gene polymorphisms and caffeine-induced anxiety [4, 5, 211]. Third, pharmacological studies with caffeine suggest the involvement of adenosine receptor in anxiety-related behavior. It is reported that adenosine has anxiolytic effects, which could be reversed by pretreatment with caffeine and theophylline [113]. Similarly, caffeine and theophylline at higher doses showed anxiogenic effects, suggesting that blockade of adenosine receptors after chronic ingestion of caffeine led to increased anxiety-related behavior. However, it should be noted that caffeine effects on anxiety are dose related: higher doses of caffeine tend to increase [83, 84, 115, 129, 192, 198] and lower doses of caffeine tend to reduce anxiety levels in humans [87, 124, 125]. The dose-dependent effect of caffeine may due to different effects of caffeine on different subtypes of adenosine receptors in anxiety. Fourth, El Yacoubi *et al.* (2000) also showed that the short-term anxiety-like effect of caffeine in mice might not be related solely to the blockade of A_2A_R, since it is not shared by A_2A_Rs selective antagonists [50]. Therefore, the role of A_2A_R in anxiety remains to be defined [27].

### 4. Depression

	The effect of the adenosine modulation on depression is complex due to the participation of several neurotransmission systems, such as dopaminergic and serotoninergic systems as well as the corticotrophin system [34, 75, 96, 179]. The involvement of adenosine in depression has also been supported by other indirect evidence showing that classical tricyclic antidepressants, such as nortriptiline, chlomipramine or desipramine, can bind to adenosine receptors and reduce the activity of ecto-nucleotidases in cortical nerve terminals [41]. Thus, the classical antidepressants also reverse the adenosine-induced immobility [112, 113]. However, the pharmacological effect of adenosine on depression is not clear yet. A series of studies showed that administration of adenosine, either peripherally or intra-cerebroventricularly, has an antidepressant effect, which involves the recruitment of adenosine receptors, the NO-cGMP system, or the opioid system [104-106]. However, other studies found that adenosine and its analogues caused depressant-like behavioral effects by increasing immobilization time in rats submitted to inescapable shocks and forced swim tests [93, 141, 142, 217].

	At the receptor level, the blockade of A_2A_Rs relieves the early stress-induced loss of synaptophysin, a synaptic marker, in the hippocampus of rats subjected to sub-chronic restraint stress [30]. A_2A_R antagonists prolong escape-directed behavior in the tail suspension and forced swim tests [49]. Additionally, A_2A_R knockout mice displayed an attenuated ‘behavioral despair’ in these two screening tests [48]. The same research group furthermore demonstrated that haloperidol (a D_2_R antagonist) prevented the antidepressant effects resulting from A_2A_Rs blockade [48, 49]. This evidence suggests a potential role of A_2A_R modulation as novel anti-depressant target.

	However, mechanisms by which the A_2A_R exerts its modulation of depression are not clear yet, but adenosine modulation of the serotoninergic system may in part be responsible [78]. For example, adenosine receptors have been shown to control the release of serotonin [156]. Furthermore, caffeine, probably *via* blockade of A_2A_R, relieves restraint-induced stress, which correlates with reduction of serotonin levels in the hippocampus [218]. Given the increasingly recognized role of neurogenesis and neuronal trophic factors in the depression-related behavior [136, 137], it is interesting to note the novel interaction between A_2A_Rs and Trk-B receptors [95], and neurotrophins, such as brain-derived neurotrophic factor (BDNF) [45, 123], which may provide another potential mechanism for the involvement of A_2A_Rs in anxiety modulation. Thus, beyond interaction with D_2_Rs, the interaction of the A_2A_R with other neurotransmitter systems, such as glutamatergic, serotoninergic, and corticotrophin system as well as trophic factors should be examined.

### 5. Schizophrenia

	Schizophrenia is a complex neuropsychiatric disorder characterized by cognitive deficits, and positive and negative symptoms [99, 150]. Almost all antipsychotics currently used in clinical practice are dopamine D_2_R antagonists, though they produce many side effects. The development of novel pharmacological targets for antipsychotics is still very limited, primarily due to the heterogeneity, lack of solid anatomical or neurochemical basis of the disorder, and lack of an adequate animal model that faithfully mimics the features of behavioral changes found in this psychiatric disorder [28, 51, 100, 101]. To date, many biochemical and neurochemical markers as well as a rather broad brain area have been implicated in the pathogenesis of diverse psychiatric disorders [11, 133, 149, 195, 197]. On the other hand, the current evaluation of the efficacy of novel antipsychotics still largely relies on the alleviation of behavioral changes that characterize schizophrenia.

	Recent progress in adenosine neurobiology supports the notion of adenosine-based therapy and the A_2A_R as a novel therapeutic target for the treatment of psychiatric disorders. The first line of evidence came from pharmacological and genetic studies showing that A_2A_R activity affects schizophrenia-like behaviors in patients. Caffeine exacerbates positive symptoms [39, 132, 138, 140, 151] of schizophrenia, whereas adenosine transport inhibitors (such as dipyridamole) and xanthine oxidase inhibitors (such as allopurinol) may be beneficial for schizophrenia [2, 3]. Intriguingly, a clinical report suggests that poorly responsive schizophrenic patients improved considerably with add-on of allopurinol [116]. Early studies found that a single-nucleotide polymorphism (SNP) of the A_2A_R gene was a candidate for a schizophrenia susceptibility gene on chromosome 22q12-13 [42, 92], but this has not been replicated by others. Furthermore, theophylline was shown to mimic deficiency of sensorimotor gating [77], as evaluated by a disturbed prepulse inhibition or P50 evoked potential found in schizophrenic individuals [167]. These observations of clinical genetics warrant further investigation.

	Second, the adenosine-hypofunction hypothesis of schizophrenia is further supported by studies from Yee *et al. *(2007) [220] using a transgenic mouse model with overexpression of adenosine kinase, causing decreased adenosine levels in forebrain. They demonstrated that subtle changes in adenosine levels in forebrain could lead to the emergence of behavioral endophenotypes implicated in schizophrenia and abnormal response to psychostimulants, i.e. amphetamine and MK-801 [220]. It is also reported that startle habituation (a measure of sensorimotor function) was reduced by A_2A_R antagonists [148] and genetic deletion of A_2A_Rs in mice [213]. The third line of evidence in supporting a role of A_2A_Rs in the pathophysiology of schizophrenia came from observations that treatment with antipsychotic drugs alter the adenosinergic system in animals and in humans [4, 135, 148, 159, 213]. It was also observed that clozapine, an atypical antipsychotic, induced c-fos expression that could be blocked by A_2A_R antagonists in rodents [159]. In addition, this clozapine-induced antipsychotic effect also affects the ectonucleotidase pathway, thus consequently modulates adenosine levels and resulting activation of A_2A_Rs [119]. In clinical studies, Martini *et al.* (2006) demonstrated an upregulation of A_2A_R in platelets from patients under treatment with haloperidol, a typical antipsychotic [135]. This study also revealed the co-expression of A_2A_Rs and D_2_Rs assembled into heteromeric complexes in human platelets. Conversely, chronic treatment with non-dopamine based atypical antipsychotic was not able to induce any significant alterations in A_2A_R equilibrium binding parameters and receptor responsiveness. In line with this finding, an upregulation of striatal A_2A_Rs has been demonstrated to occur in schizophrenia patients with antipsychotic treatment [4]. Noticeably, the increased A_2A_R density correlated with the dose of antipsychotics in chlorpromazine equivalents, which suggests a role of A_2A_Rs in the molecular effects of antipsychotic drugs.

	The fourth line of evidence came from molecular studies suggesting a modulatory role of A_2A_Rs as a fine-tuner in re-balancing an impaired glutamatergic-dopaminergic communication. Regarding dopaminergic function, the antagonistic interaction of A_2A_R-D_2_R in the striatum suggested anti-psychotic behavior in schizophrenia by A_2A_R agonist to function as a dopamine receptor antagonist. The activation of A_2A_Rs can reduce D_2_R affinity and function, which may potentially underlie the antipsychotic-like profile of adenosine agonists [56], the hyperdopaminergic effect of caffeine [53, 56] and the exacerbation of psychotic symptoms by caffeine in schizophrenic patients [132]. More data discussed in other reviews suggested the relationship between hyperdopaminergic transmission and unbalanced adenosinergic modulation in the striatum [82, 120, 187, 202]. These observations support the possibility that the manipulation of A_2A_Rs (by activation of A_2A_R) may help restore an adequate dopaminergic signaling. Regarding glutamatergic function, A_1_R and A_2A_R agonists have both been shown to prevent behavioral and EEG effects induced by NMDAR antagonists [166, 193]. In an NMDAR hypofunction model of schizophrenia [157], the function of NMDARs could be modified by both A_1_R and A_2A_R activities [40, 76, 172, 208, 216]. Furthermore, both A_1_Rs and A_2A_Rs control the evoked release of glutamate in striatum [24, 177]. Conversely, the activation of the NMDAR increases the adenosine tone [139], while inhibition of the NMDAR reduced adenosine release [43]. Importantly, the psychostimulant effects of NMDAR antagonists are largely abolished by genetic inactivation or pharmacological blockade of A_2A_Rs [176, 188]. These studies suggest that modulation of A_2A_Rs may re-balance the hypofunction of NMDARs in models of schizophrenia. As reviewed in the above sections, the existence of heteromeric A_2A_R-D_3_R and A_2A_R-mGluR5 receptor complexes may also strengthen the potential modulation of A_2A_R on schizophrenia therapy [206].

	However, the effect of adenosine modulation on psychiatric disorders is likely more complex, with involvement of different neurotransmitter systems in various brain regions. For example, we recently demonstrated that striatal deletion of A_2A_Rs enhances the actions of psychostimulants, whereas deletion of A_2A_Rs in forebrain (including striatum, cortex and hippocampus) attenuates the effect of psychostimulants. These data suggest that striatal A_2A_Rs and extra-striatal A_2A_Rs exert different effects on psychomotor activity. Thus, adenosine-based psychopharmacological therapy may rely on the status or degree of dysfunction in other neurotransmitters, or spatial targeting of adenosine agonists/antagonists, or drug specificity, selectivity, dosage and paradigm.

### 6. Learning and Memory

	Several recent pharmacological and genetic studies suggest a potential modulatory role of brain A_2A_R activity on learning, memory, and other cognitive process [34, 181]. For example, local administration of A_2A_R agonists into the posterior cingulate cortex impaired memory retrieval in rats [158]. Conversely, the A_2A_R antagonist SCH58261 and caffeine have been shown to improve social recognition memory [169] and improve memory performance in rodents through different tasks [206]. Genetic inactivation of the A_2A_Rs enhanced spatial recognition memory and novelty exploration in Y-maze testing in mice [212]. Recently, two studies demonstrated that both pharmacological blockade and genetic inactivation of A_2A_Rs attenuated β-amyloid-induced memory loss [29, 37]. The above results suggest that the A_2A_R activity can modify the spatial memory process in rodents. 

	On the other hand, working memory primarily depends on the integrity of prefrontal cortical function [80] and is critical to human reasoning and judgment, which is at the core of pathophysiology for many neuropsychiatric disorders such as Alzheimer’s disease [8, 107, 126] and schizophrenia [81]. The control of working memory by A_2A_Rs [169] is supported by several elegant behavioral studies showing an impact of caffeine [168]. Recently, transgenic overexpression of A_2A_Rs in cortex has been shown to impair spatial working memory in radial maze, repeated trials of Morris water maze and objective recognition tests [79]. In agreement with this finding, we recently observed (unpublished data) that genetic inactivation of A_2A_Rs significantly improved working memory; furthermore, the improved working memory was selective for this specific short-term memory whereas the performance of spatial reference memory and the memory retention after prolonged training was largely indistinguishable between A_2A_R knockout mice and their WT littermates. These results suggest a selective modulatory role of A_2A_R activity in working memory. 

## CONCLUDING REMARKS

	In this review, we have described the role of adenosine A_2A_ receptor-driven interactions with other neurotransmitter systems, at multiple levels of psychopharmacology, from the molecular basis of receptor-receptor cross-talk, to pharmacological and genetic manipulations of A_2A_R activity, and the alteration of neuropsychiatric phenotypes in psychostimulant addiction, anxiety and depression, schizophrenia and learning and memory. Based on the literature to date, the A_2A_R is involved in multiple receptor-receptor interactions, multiple neurotransmissions and multiple neuropsychiatric disorders. In particular, it tightly interacts with two main neurotransmitter systems, the dopaminergic and glutamatergic signaling pathways, with implications for a wide range of psychiatric behaviors and several psychiatric disorders. Hence, A_2A_Rs are ideally positioned as a fine-tuner, providing integrated effects between glutamatergic and dopaminergic signaling, and may represent a novel neuropsychopharmacology target.

	Despite its attractive therapeutic potential, several concerns need to be introduced, when evaluating the putative role(s) of A_2A_Rs in psychopharmacology. First, since adenosine system works *via* neuromodulation, the modulatory ability of the adenosine system (including the A_2A_R) may depend on and may intricately be linked with the activity of other “potent” neurotransmission systems, i.e. dopaminergic, glutamatergic and serotoninergic systems. Second, a primary role of the adenosine neuromodulatory system seems to be maintenance of homeostasis or promotion of the adaptation of multiple neurotransmitter systems in the brain. Thus, adenosine, and A_2A_Rs in particular, seem to curtail extremes (i.e. over-stimulation or under-stimulation) of these neurotransmitter systems in the brain. A_2A_R-based modulation may largely be exerted, once disequilibrium of neurotransmitter systems occurs. Third, extracellular adenosine may act at A_2A_Rs and A_1_Rs with globally opposite functions, or may act at the A_2A_R in different brain regions with its differential action to exert modulating effects. The balanced outcome of adenosine actions may be in part controlled by neuroadaptation or maladaptation of neurotransmission, by which it exerts its effect and may in part depend on the preferential sites of pharmacological reagent activity.
